# Progress in Surgery

**Published:** 1898-05-07

**Authors:** 


### PROGRESS IN SURGERY.

### NEW GROWTHS.

(*Continued from page 64.*)

**The Treatment of Inoperable Malignant Tumours.**—(1) By Coley's Fluid: In September, 1896, Coley published an account of 90 cases of sarcoma treated in this way. In 13 of these the tumours disappeared entirely, and in nine of these there had been no recurrence. Thirty-three of the patients improved. All the cases, with one exception, were verified microscopically. Exactly half the cases showed no change. Spindle-cell sarcomata were found to be much the most amenable. Mansell Moullin[7] has tried the treatment in nine cases. In three the growths had practically disappeared, and in two they had diminished considerably in size. The nature of the tumours in the cases apparently cured had not been verified microscopically. Dr. Coley[8] records another case in 1897 in which a spindle-celled sarcoma of the abdominal wall was cured. Just above the symphysis pubis, and extending nearly to the umbilicus, to the right of the median line two inches, and to the left one inch, was a tumour apparently located in the abdominal wall. It was first explored, and a portion removed for examination. It was found to be both intra and extra-peritoneal, and considered to be irremovable. The treatment lasted from January 19th to March 10th, during which time 30 injections were given, producing six chills. The patient was discharged perfectly cured, no trace of induration even being discoverable. (2) By Injections of Alcohol: A case of scirrhus of the breast treated for ten weeks with repeated multiple injections of a 40 per cent. solution pure alcohol in water is recorded by Dr. Yeates.[9] The enlarged and infiltrated breast and axillary glands dwindled away until they became scarcely perceptible. The patient unfortunately then died from secondary hepatic infiltration. It looks, however, as if the injections had caused the rapid local improvement by inducing atrophic processes. (3) By Injections of Pure Yeast: Cattel[10] summarises the results of this treatment in the practice of Dr. De Backer, of Paris, as follows: A hundred cases were treated, and all are said to have benefited. An injection of 1 c.c. of pure yeast is made in the neighbourhood of the cancerous tumour; this injection is repeated two, three, or four times (rarely more), at intervals of two days. An amelioration is seen at once. No untoward effects are produced unless impure commercial yeast is used. The action of the ferment is explained in the theory that the neoplasm contains glycogen. The glycogen, essential to the growth of the tumour, is destroyed by the fermentation of the yeast, and thus the growth of the tumour is retarded. The result is supposed to be favoured by the production of alcohol in a nascent state. (4) By anti-streptococcic serum: Zirmacki[11] has thus treated 20 cases of inoperable malignant new growth. The serum was obtained from goats and horses, the immunisation being carried out by the injection of living cultures. Six cases were of sarcoma and fourteen of carcinoma. In no case was the treatment efficacious. The author cannot confirm the statement that the ulcerating surface rapidly cleans.

**Primary Carcinoma of the Pleura.**—Benda[12] records a case of this disease. The patient, a workman, aged fifty-four years, was first seen ten months before death. For a year previous to this he had complained of pain in the left side of the chest. The patient was aspirated sixteen times, clear fluid in large quantities being withdrawn each time. Finally a rib was resected and the pleura opened and drained. Suppuration followed, and four weeks after the operation he died with pyæmic symptoms. A post-mortem showed that the entire left pleura was studded with nodular masses scarcely reaching a cherry in size. Only in one or two places had the nodules shown any tendency to invade the lung. The diaphragmatic muscle was somewhat infiltrated. There were no other growths in the body. Microscopically the tumour was found to be made up of cells of epithelial type in a stroma. An interesting point was that there was a gradual transformation in shape of the superficial endothelial cells to the epithelial-like tumour cells. Many pathologists believe that tumours of the serous cavities resemble carcinomata, but according to Benda no cases have been recorded in support of this view.

**The Supposed Increase of Cancer Mortality.**—Dr. Newsholme[13] has recently written on this subject. He alludes to the explanation which attributes the apparent increase of cancer mortality to the lowered general death-rate. If more persons live to older ages, more will die from the diseases of degeneration, cancer among the rest. But this does not imply any addition to the cancer death-rate at these ages, when the numbers living at the age are considered. Newsholme shows that this explanation is by no means sufficient to explain the increased cancer mortality. Two explanations remain. Either there has been a real increase of the mortality, or the apparent increase is the result of a more accurate diagnosis and certification of death. It is this latter view that Newsholme holds.

**Carcinoma of the Breast.**—The Royal Medical and Chirurgical Society has devoted three meetings to a discussion on the operative treatment of cancer of the breast.[14] Mr. Shield read a paper on "Immunity and Latency" after these operations. A number of cases in which no recurrence had followed the old operation were tabulated, and also a number, sixty-four, in which though recurrence took place the patients lived for long periods. Thus, the results of the old operation did not appear to be so universally bad as generally stated. Mr. Bryant made the interesting remark that in some cases of recurrent carcinoma he had noticed many cutaneous tubercles, and in one such case, of which he made drawings at the time, the tubercles after existing more than six months disappeared, and he had seen such a disappearance in eight or ten instances. Mr. Barwell laid stress upon not removing healthy skin as tension in the wound hindered rapid healing and made recurrence more likely. Mr. Butlin stated that he had lately employed Halsted's method, and he thought it would still further reduce the number of recurrences. Sir Thomas Smith was inclined to think it possible that the removal of healthy glands might precipitate the spread of the disease. He thought the chief factors in bringing about immunity after operation were the stage at which the operation was performed, the type of the growth, and the soil in which it had grown. Mr. Nunn pointed out that recurrence in the opposite mamma was not very rare, and that Copeman found it in 19 per cent. of the cases of mammary cancer which he had collected. Mr. Howard Marsh stated that he did not remove the pectoral muscle, as Halsted had recommended, as he knew of no case in which recurrence took place in the muscle. This removal of the muscle increased the severity of the operation and had dangers of its own. The patient suffered from much shock and several had died from that cause. Mr. Bennett said he had seen cancer recur in the muscle, but it was rare. Mr. Cheyne objected to the modern complete operation being called Halsted's. The investigations had been made and the rules for operation laid down by Heidenhain and Stiles.

Before the College of Physicians of Philadelphia, Mr. G. B. Wood[15] recently compared Kocher's method of amputating the breast for carcinoma and Halsted's. The results of Kocher's operation, he urged, were at least equally good, and less time and skill were required for its performance. The salient points of difference between them are: First, the incisions are different. In Kocher's, the outer end of the lower incision encircling the breast is carried along the posterior fold of the axilla, up over the belt formed by the pectoralis major, stopping at the clavicle. This, it is maintained, allows a free entrance to the armpit, and does not take as much time as the dissection backwards of the triangular flap of Halsted. By Kocher, the axilla is cleaned from above downward and inward toward the chest, while Halsted makes the dissection from within upward and outward. The vessels supplying the axilla begin above and run downward, and it is easier to dissect out a vessel from the base towards the periphery than in the other direction. But the chief saving of time is in the removal of the pectoral muscle, it being much easier to strip this muscle downward and inward off the chest, and then cut through the origins, than it is to dissect off each rib attachment separately. By beginning as advised by Kocher, the annoyance of having a large loose mass of tumour, fat, and muscle to take care of during the dissection of the axilla is avoided. Kocher recommends a temporary resection of the clavicle if the glands above that bone are infected, and in case of infiltration of the fascia immediately covering the ribs and intercostal muscles he would resect a piece of the chest wall.

[7] Lancet, Feb., 1898. [8] Annals of Surgery, Aug. [9] Brit. Med. Jour., Sept. [10] Internat. Med. Mag., Nov. [11] Brit. Med. Jour., Jan., 1898. [12] Amer. Jour. Med. Science, Oct. [13] Brit. Med. Jour., Oct. [14] Lancet, Jan. and Feb., 1898. [15] Annals of Surgery, Feb.

